# Thalamocortical Projection Neuron and Interneuron Numbers in the Visual Thalamic Nuclei of the Adult C57BL/6 Mouse

**DOI:** 10.3389/fnana.2018.00027

**Published:** 2018-04-12

**Authors:** Marian Evangelio, María García-Amado, Francisco Clascá

**Affiliations:** Department of Anatomy and Neuroscience, School of Medicine, Autonomous University of Madrid, Madrid, Spain

**Keywords:** thalamus, lateral geniculate nucleus, pulvinar nucleus, lateral posterior nucleus, thalamic interneurons, stereology

## Abstract

A key parameter to constrain predictive, bottom-up circuit models of a given brain domain is the number and position of the neuronal populations involved. These include not only the neurons whose bodies reside within the domain, but also the neurons in distant regions that innervate the domain. The mouse visual cortex receives its main subcortical input from the dorsal lateral geniculate nucleus (dLGN) and the lateral posterior (LP) complex of the thalamus. The latter consists of three different nuclei: lateral posterior lateral (LPL), lateral posterior medial rostral (LPMR), and lateral posterior medial caudal (LPMC), each exhibiting specific patterns of connections with the various visual cortical areas. Here, we have determined the number of thalamocortical projection neurons and interneurons in the LP complex and dLGN of the adult C57BL/6 male mouse. We combined Nissl staining and histochemical and immunolabeling methods for consistently delineating nuclei borders, and applied unbiased stereological cell counting methods. Thalamic interneurons were identified using GABA immunolabeling. The C57BL/6 dLGN contains ∼21,200 neurons, while LP complex contains ∼31,000 total neurons. The dLGN and LP are the only nuclei of the mouse dorsal thalamus containing substantial numbers GABA-immunoreactive interneurons. These interneurons, however, are scarcer than previously estimated; they are 5.6% of dLGN neurons and just 1.9% of the LP neurons. It can be thus inferred that the dLGN contains ∼20,000 and the LP complex ∼30,400 thalamocortical projection neurons (∼12,000 in LPL, 15,200 in LPMR, and 4,200 in LPMC). The present dataset is relevant for constraining models of mouse visual thalamocortical circuits, as well as for quantitative comparisons between genetically modified mouse strains, or across species.

## Introduction

Predictive, bottom-up digital models of neural circuits are promising new tools for deepening our mechanistic understanding of brain structure and function. Starting from sparse (yet spatially and quantitatively accurate) experimental data about cell numbers and position, dendritic and axonal morphologies and known synaptic connections and conductances, these models are able to reconstruct algorithmically the cellular and synaptic relationships of complex brain circuitry. Such models are, by definition, open to increasing refinement with the addition of new data, and can be used, for example, to investigate *in silico* the dynamic behavior of brain circuits at different scales, or even to predict unknown synaptic relationships (Hill and Tononi, 2004; Markram et al., 2015; Hawrylycz et al., 2017).

A key parameter to constrain the bottom-up model of a given brain domain are the cell numbers and spatial position of the neuron populations participating in its circuits. The populations that should be accounted for are not only those whose cell bodies reside within the domain, but also the neurons in distant brain regions whose axons innervate the region being studied. For example, the thalamocortical projection neurons must be included in a model of cortical circuits (Markram et al., 2015; Hawrylycz et al., 2017). Moreover, since neuron population numbers can vary widely between species and strains (Seecharan et al., 2003; Herculano-Houzel et al., 2011), the data source should be, as much as possible, consistent also regarding these parameters.

Anatomical and functional studies of the mouse visual thalamocortical system have historically lagged behind those of rats, cats, or macaques (Chalupa and Williams, 2008). However, with the growing relevance of the mouse as a model for systems neuroscience, the organization of its visual cortex and thalamocortical pathways has in recent years become intensively investigated (Seabrook et al., 2017; Zhou et al., 2017 for reviews). The mouse primary and association cortical visual areas receive most of their thalamic inputs from the dLGN and the LP complex (Tohmi et al., 2014). The latter consists of three main nuclei: lateral posterior lateral (LPL), lateral posterior medial rostral (LPMR), and lateral posterior medial caudal (LPMC), each exhibiting specific patterns of connections with the various visual cortical areas (Wang and Burkhalter, 2007; Seabrook et al., 2017).

More than twofold differences in neuronal numbers in the same forebrain structures have been demonstrated between mouse strains (Seecharan et al., 2003); thus, numerical data for modeling should be derived, as much as possible, from the same mouse strain. The C57BL/6 inbred strain is the most widely used genetic background for genetically modified mice for use as models of human disease. All the modern atlasing efforts on the mouse brain, both anatomical (Lein et al., 2007; Paxinos and Franklin, 2012), gene expression (Lein et al., 2007; Ng et al., 2010), and connectomic (Markram, 2012; Oh et al., 2014; Economo et al., 2016; Hawrylycz et al., 2017) have chosen C57BL/6 as their model.

The number of thalamocortical projection neuron numbers in the visual thalamic nuclei of the adult C57BL/6 mouse has not been determined. Cell numbers in LP or its subdivisions have never been counted in any mouse strain. Total neuron numbers in the dLGN have been estimated for the adult (Seecharan et al., 2003) and 10-day-old (Dursun et al., 2013) C57BL/6 mice. None of these studies, however, distinguished thalamocortical projection neurons from the GABAergic interneurons which, according to published estimates, might represent a sizable fraction of the dLGN neurons (up to 15–20% according to Arcelli et al., 1997; see also Li et al., 2003). It is to be noted, that these estimates were not conducted using unbiased methods, and thus the actual numbers of thalamocortical projection neurons in the dLGN of C57BL/6 mice remain unclear.

In the present study, we set out to determine both the number of thalamocortical projection neurons and interneurons in the LP complex and dLGN of the adult C57BL/6 male mouse. We combined Nissl staining and histochemical and immunolabeling methods for consistently delineating nuclei borders, and applied unbiased stereological cell counting methods. Interneurons were identified using GABA immunolabeling.

## Materials and Methods

### Animals

All experimental procedures involving live animals were carried out in the Autonoma de Madrid University, in accordance with the European Community Council Directive 2010/63/UE (official authorization from Consejeria de Agricultura y Ganadería de la Comunidad de Madrid, PROEX175/16). A total of 15 male C57BL/6 mice of 60–85 days of age (25–30 g) were used. Animals were bred in our University animal facilities (register code ES280790000097). Genetic background consistency and stability was ensured by the periodic inclusion in the stock of new breeding animals purchased from Jackson Laboratories (Bar Harbor, ME, United States). Animals were housed in extra-large cages with food and water *ad libitum* under a 12 h light/dark cycle. All animals were born from different progenitors. At the time of sacrifice, the animals’ eyes and vibrissae were complete and healthy on inspection.

### Perfusion and Tissue Processing

Following a lethal dose of sodium pentobarbital (0.09 mg/g, i.p.) and a thoracotomy, animals were perfused through the left ventricle with saline solution (0.9%) followed by paraformaldehyde 4% in 0.1M phosphate buffer (PB) at 4°C, for 30 min. In the cases prepared for GABA immunohistochemistry, 0.2% glutaraldehyde was added to the perfusion solution. Brains were then removed from the skull and postfixed by immersion for 24 h in the perfusion solution and subsequently cryoprotected by embedding for 24 h at 4°C in a sucrose solution (30% in PB 0.1M).

Two series of parallel sections (50 μm thickness) were cut either in the coronal (*n* = 10) or sagittal plane (*n* = 2) on a freezing microtome (Leica Microsystems, Nussloch, Germany).

In five coronally sectioned brains, one series was processed for Nissl staining with cresyl violet while the other series was stained histochemically to reveal AChE activity (Geneser-Jensen and Blackstad, 1971). In two other brains (one cut coronally and other cut sagittally), the series were immunostained either for calbindin D28k (CB) or CR. Two further brains were sectioned likewise and then processed for cytochrome-oxidase histochemistry (Wong-Riley, 1979) or VGluT2 immunolabeling. Finally, the sections from three brains were immunostained for GABA, or kept as a reserve.

For VGluT2, CR, and CB immunolabeling, sections were first incubated in 0.1% oxygen peroxide (20′) and 2% Triton PB. Sections were then incubated, free-floating, in the corresponding primary antibodies (**Table 1**). After washing in PB, labeling was intensified with a secondary biotinylated antiserum and then visualized using glucose oxidase and avidin–biotin peroxidase complex (Vectastain Elite, Vector labs) with nickel sulfate 0.1% (Sigma) for enhanced contrast (Shu et al., 1988). Omission of primary antibody, used as control for the specificity, yielded no cell labeling.

**Table 1:** Antibodies used in the study.

Antibody	Origin	Source	Dilution	Incubation
vGluT2	Guinea Pig	Merck Millipore	1:2000	24 h/RT
CR	Rabbit	Swant	1:5000	24 h/RT
CB	Mouse	Sigma-Aldrich	1:2000	48 h/4°C
GABA	Rabbit	Sigma-Aldrich	1:500	48 h/RT
Biotinylated-anti-Rabbit	Goat	Sigma-Aldrich	1:100	2 h/RT
Biotinylated-anti-Mouse	Goat	Vector Laboratories	1:500	2 h/RT
Biotinylated-anti-Guinea Pig	Goat	Vector Laboratories	1:200	2 h/RT

For the GABA immunolabeling, pre-treatment was instead carried out using 10% oxygen peroxide and 10% methanol in Tris-buffered saline (TBS) pH 7.6, 0.1M. In addition, we applied an antigen retrieval protocol consisting of a rising in sodium citrate buffer pH6.0 at 90°C for 60 min followed by slow cooling for 60 min in the same solution. Incubation in primary (48 h, room temperature) and secondary antisera was then carried out in phosphate-buffered saline (PBS, see **Table 1**). Omission of primary antibody, used as control for the specificity, yielded no cell labeling.

Finally, sections were mounted on gelatin-coated glass slides, dehydrated in graded alcohols, defattened in xylene, and coverslipped with DePeX (Serva).

### Tissue Imaging and Delineation of Nuclear Boundaries

Complete series of coronal and sagittal Nissl-stained sections were imaged at 10× on an Eclipse 600 microscope fitted with a DXM1200 camera (Nikon, Japan). High-resolution panoramic images were obtained using a precision motorized stage (ProsCan, Prior Scientific Instruments, Cambridge, United Kingdom) driven by the NIS imaging software package (Nikon) that is able to control image acquisition and stage motion.

Nuclear boundaries were delineated by overlaying the microscope images from adjacent sections stained for Nissl, AChE, and CyO histochemistries, and/or VGluT2, CR, and CB inmunostainings with Canvas 15 software (ACD Systems, Victoria, BC, Canada). Fiber tracts and differences in staining intensity were used to establish boundaries. The borders of dLGN and LP could be delineated with confidence on Nissl-stained sections; we used them for cell counting estimations in the individual brains analyzed (**Supplementary Figure S1**). In contrast, even from the comparison of multiple stainings, delineation of borders between LP subdivisions was less precise, and remained open to observer bias. For this reason, we did not directly count cell numbers in each LP subdivision on the individual brains. As an approximation, we inferred these numbers by extrapolating from the percentage that each subdivision’s volume (measured in a CR/VGluT2-stained series, see Results) represents on the total LP volume.

### Stereological Estimations

#### Microscope Setup

Stereological analyses were carried out with an Olympus BX61 light microscope (Olympus, Japan) equipped with a microcator Heidenhain MT12 (0.5 μm resolution; Heidenhain, Traunreut, Germany), an X-Y-Z high precision motorized microscope stage (ProScan II, Prior Scientific, Cambridge, United Kingdom), and an Olympus DP-71 digital camera connected to a computer running the NewCAST stereology software package (Visiopharm, Hørsholm, Denmark). The objectives used 4× PlanApo to trace the contours of the thalamic nuclei to estimate the volume with the Cavalieri method; and the 100× PlanApo 1.4 N.A. oil immersion lens (Olympus, Tokyo, Japan) to perform the neuron counting.

#### Reference Volume Estimation

This method has been described in detail elsewhere (García-Amado and Prensa, 2012) and is dealt with only briefly here. Volume estimations for each nuclei (V) were obtained by means of the Cavalieri principle (Gundersen and Jensen, 1987) over one out of two Nissl coronal sections. Briefly, we used an area per point of 0.14 mm^2^ and a distance between analyzed sections of 100 μm. Considering that areal shrinkage suffered by the tissue sections after Nissl staining and mounting is negligible (Blasco et al., 1999), this method delivered reliable volume estimations of fixed mouse brains.

#### Estimation of Neuron Numbers With the Optical Fractionator

To reliably identify neurons on Nissl-stained tissue, we used the following morphological criteria (Bezchlibnyk et al., 2007; Konopaske et al., 2007): neurons were identified because of their large size, nuclear euchromatin, patent nucleolus and surrounding cytoplasm (**Supplementary Figure S1**). Glial cells were readily distinguishable because of their small size, presence of heterochromatin in the nucleus and lack of visible cytoplasm. Endothelial cells were identified because of the curved shape of their nuclei, and the fact that they are usually included in the wall of a patent blood vessel. For total neuron counting on the Nissl stained material, we considered the nucleolus as the counting unit.

Interneuron counting was carried out on the GABA-immunostained sections. In this case, we used the nucleus equator, as the nucleolus was not clearly visible with the GABA staining (**Supplementary Figure S2**).

To calculate the numbers of total neurons (*N_neu_*) and interneurons (*N_IN_*), we applied an optical fractionator design (West and Gundersen, 1990). Thus, every region was systematically, uniformly and randomly sampled under a 100× Plan-Apochromatic oil immersion objective. In a pilot study, we first established the proper sampling parameters for counting at least 100 neurons or more of each nucleus in each animal, which was enough to get a coefficient of error (CE) below 0.1.

Neurons numbers were estimated with the equation:

$$\begin{array}{c} N_{neu}=\frac{1}{ssf}\cdot \frac{1}{asf}\cdot \frac{1}{hsf}\cdot \sum Q^{-} \end{array}$$

*ssf* (section sampling fraction, 1/2), *asf* (area sampling fraction, 0.044 for total neurons and 0.64 for interneurons), *hsf* (height sampling fraction), and ∑ Q^-^ is the number of cells counted in every region. Finally, the *hsf* value comes from $\frac{h}{{\bar{t}}_{Q^{-}}}$ where *h* is the disector height (8 μm for total neurons/10 μm for interneurons) and ${\bar{t}}_{Q^{-}}$ is the weighted mean value of the section thickness measured with the microcator (Bermejo et al., 2003). ${\bar{t}}_{Q^{-}}$ value reports information about the variability in thickness measurements observed within a single section and between sections indicating that the *z*-axis compression in frozen sections is uneven, which results in a more reliable number estimation. For that purpose, section thickness was measured in the *z*-axis in one out of three disector containing at least one neuron. We also considered a defined upper and lower guard zones from the disector (3 μm upper and variable lower guard zones) in order to avoid the loss of particles bias at the sectioning surface due to the “lost caps” effect and incorrect cell identification (Andersen and Gundersen, 1999; Baryshnikova et al., 2006).

#### Precision of the Stereological Estimates

We calculated the CE due to the sampling method for both volume and neuron number estimates using the equations detailed in Gundersen et al. (1999). The mean CE for all cases (${\bar{t}}_{Q^{-}}$) was obtained with equation below where *n* is the number of studied cases. In cases of smaller nuclei such as LPMC higher CEs were obtained in the cell counts, but increasing the number of sections or the sampling intensity in these small regions to achieve lower CEs would not be justified.

$$\begin{array}{c} \hat{CE}=\sqrt{\frac{\sum CE^{2}}{n}} \end{array}$$

### Statistical Analysis

Mean and deviation were calculated for all the estimations. Differences between right and left thalami were analyzed with the SPSS 23 software (IBM SPSS Statistics, Chicago, IL, United States) by using a paired *t*-*Student* test in the cases where samples were adjusted to normality. For non-normal distributions, we used the Wilcoxon test in order to determine these side discrepancies. Significant differences were considered when the *p*-value was lower than 0.05.

## Results

### Delineation of the dLGN and LP Complex Nuclei

Establishing a consistent delineation of the tissue volume to be measured is a key methodological requirement for the accurate quantification of neuronal numbers and volumes. In the present study, we adapted to mice the delineation criteria proposed by Kamishina et al. (2009) and Masterson et al. (2009) for the rat dLGN and LP complex. Below, we make these criteria explicit.

**FIGURE 1:**
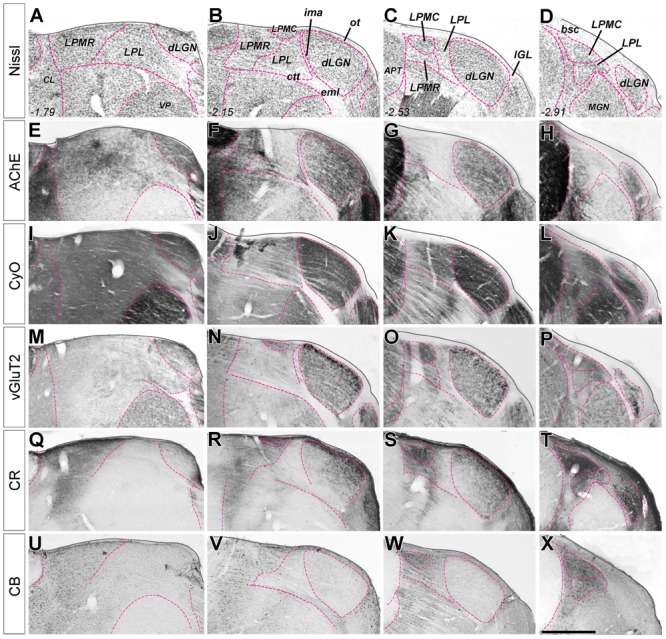
Delineation of visual thalamus nuclei from the combination of multiple stains on coronal sections. **(A–D)** Nissl-stained coronal sections of mouse visual thalamus. Sections are ordered in the row from rostral (left) to caudal (right). Distance caudal (–) to Bregma (in mm) is indicated in the lower left corner for each level. All nuclei boundaries (purple dashed line) are drawn here on the images, and the nuclei identified, for reference. In **(E–X)**, only the boundaries that are revealed by the staining at each coronal level are marked with the purple line. **(E–H)** Parallel coronal sections stained with acetylcholinesterase histochemistry. **(I–L)** Parallel coronal sections stained with cytochrome C-oxidase histochemistry. **(M–P)** Parallel coronal sections immunostained for vesicular glutamate transporter type 2. **(Q–T)** Parallel coronal sections immunostained for calretinin. **(U–X)** Parallel coronal sections immunostained for Calbindin 28K. Scale bar = 50 μm.

The visual nuclei constitute the caudo-lateral portion of the mouse thalamus. They are dorsally wrapped by the pia mater and/or by the fibers of the brachium of the inferior colliculus and the optic tract; ventrally, they are separated from the ventral posterior and the posterior thalamic nuclei (Po) by the dorsal part of the superior thalamic radiation and the corticotectal tract. The latter crosses the thalamic gray matter as several small parallel bundlets interposed between LP and Po (**Figure 1B**). We took the most ventral of these bundlets as the border between LP and Po. The medial border of the LP complex is formed by the internal medullary lamina and the central lateral/posterior limitans thalamic nuclei (Paxinos and Franklin, 2012).

**FIGURE 2:**
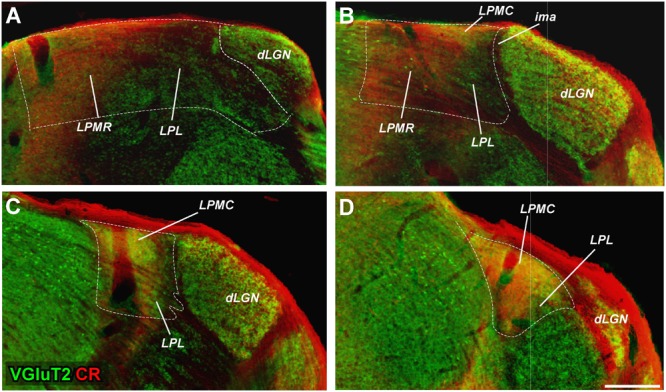
Delineation of LP subdivisions from the overlay of VGluT2 and CR immunostaining images. **(A–D)** Images from adjacent sections immunostained, respectively, for VGluT2 and CR immunolabeling (colored in red and green, respectively) are overlaid. This analysis reveals that LPMR is VGluT2-poor and CR-rich (red in the merged images), LPL is VGluT2 and CR-poor (green), and LPMC is both VGluT2 and CR rich (yellow).

Delineation of LGN and of the LP complex is straightforward in Nissl-stained sections (**Figures 1A–D**). Borders between the various nuclei within LP, however, are less apparent. These can be traced with relative confidence from the comparison of the staining patterns produced by several techniques. **Figure 1**, compare, for the same coronal levels, the staining patterns for Nissl, AChE and CyO histochemistry, and vGluT2, CB or CR immunolabeling in four matching coronal levels. **Figure 2** shows how the combined information from vGluT2 and CR stainings are particularly informative for delineating the LP subnuclei.

#### Dorsal Lateral Geniculate Nucleus (dLGN)

**FIGURE 3:**
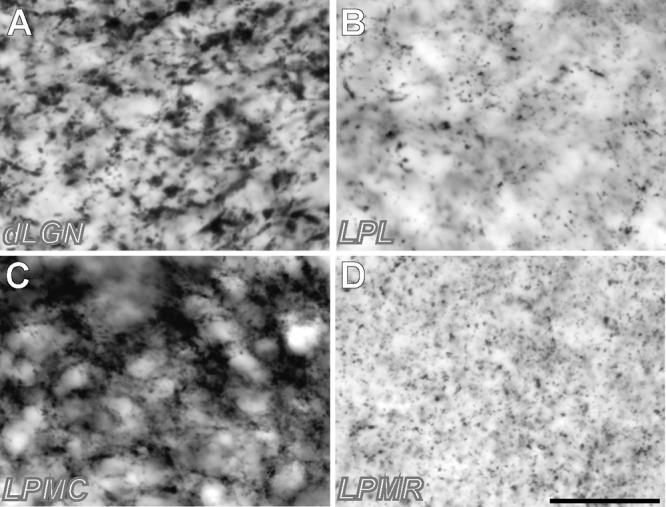
High-magnification samples of VGluT2 neuropil labeling in different thalamic visual nuclei. **(A)** Sample from dLGN. **(B)** Sample from LPL. **(C)** Sample from LPMC **(D)** Sample from LPMR. Note that the vGluT2 corpuscles (putative neurotransmitter vesicle pools in axon terminals) are larger and more abundant in LPMC and dLGN. Samples were taken from the regions pointed with a yellow asterisk in **Figure 2**. Scale bar = 25 μm.

Rostro-caudally, the dLGN extends from approximately from Bregma –1.60 to –3.0 mm. In addition to its dense cell packaging in Nissl-stained sections, dLGN borders are also sharply delineated by its heavy neuropil staining for AChE and vGluT2. The latter two techniques are also useful to delineate, because of their low or absent staining, the IGL and the thalamic intramedullary lamina/external capsule. Under high-magnification optics, the VGluT2 labeling in dLGN can be seen to consist mostly of large (>2 μm diameter) clumps (**Figure 3A**), probably corresponding to neurotransmitter vesicle pools inside large axon terminals from retinal ganglion cells (Dursun et al., 2013; Bopp et al., 2017).

#### Lateral Posterior Lateral Nucleus (LPL)

This nucleus forms a band extending from Bregma –1.80 to Bregma –3.0 along the medial border of dLGN intramedullary thalamic lamina. The line separating it from the other LP complex nuclei can be traced along a rather abrupt change in CR-staining (Zhou et al., 2017). In addition, compared to other LP complex nuclei, LPL is characterized by the virtual absence of CB-positive cell somata. The fiber bundles of the corticotectal tract mark the ventral limit of LPL. Some authors (Paxinos and Franklin, 2012) have distinguished a rostral retino-recipient and a caudal tecto-recipient subdomains; however, we were not able to detect this border consistently with our techniques, and thus we delineated and measured the LPL as a whole.

#### Lateral Posteromedial Caudal (LPMC)

This nucleus is dorsally located within the LP complex, and can be seen from coronal levels –2.40 to –3.20 mm from Bregma. Its medial border with the dorsal APT is patent with all stainings. It can be readily separated from LPL by its heavy staining for CR and numerous CB-positive cell somata, and from the LPMR by its heavy staining for VGluT2. As in dLGN, this VGluT2 staining consists mostly of large, densely packed putative synaptic boutons (**Figure 3C**), presumably corresponding in this case to terminals of axons from the superficial layers of the superior colliculus (Zhou et al., 2017).

#### Lateral Posteromedial Rostral (LPMR)

This nucleus forms the medial and rostral portion of LP from Bregma –1.6 to –2.6 mm. As indicated above, its defining traits are a heavy CR staining and a faint VGluT2 staining. The latter consists almost exclusively of small (<1.5 μm) boutons, which might correspond to retinal terminals (Morin and Studholme, 2014). Its medial border with the central lateral nucleus is difficult to delineate with precision; we took the sharp decrease in AChE-stained neuropil of LPMR compared with the central lateral nucleus (**Figures 1E,D**).

### Volume of dLGN and LP Complex Nuclei

**FIGURE 4:**
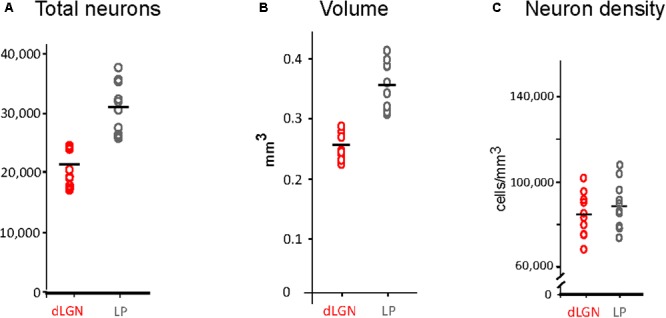
Stereological estimations of volume, total neuron number and density of dLGN and LP. **(A)** Total number estimations. Data points correspond to measurements in 10 cerebral hemispheres (five animals). Mean values are represented by a horizontal black line. **(B)** Volume estimation in mm^3^. Conventions as in **(A)**. **(C)** Estimations of neuronal density (cell/mm^3^).

Thalamic nuclei volume estimations are shown in **Figure 4B** (see also **Supplementary Table S2**). The LP complex volume ranged between 0.31 and 0.41 mm^3^ and was 30% larger than the dLGN, which ranged between 0.22 and 0.28 mm^3^. A significant difference was detected between the right and left dLGN (*p* = 0.039), the right side being smaller (**Supplementary Table S2**).

The LP subdivision volumes were measured in only two series of sections with alternate VGluT2 and CR stainings. Given the poor histological definition of the subdivisions boundaries, these volumes are given only as an approximation. In any case, it is clear that the LPMR is the largest subdivision, occupying about 49% of LP, whereas the LPL and LPMC comprise about 37% and 14%, respectively.

### Neuron Numbers in dLGN and LP Complex

Neuron number estimations in the dLGN and the LP complex are summarized in **Figure 4A** and **Supplementary Table S1**. The dLGN contains 21,193 ± 2,264 SD total neurons, while the LP contains 31,054 ± 3,920 SD total neurons. No significant differences were found for the number of neurons in each hemisphere, neither in the dLGN or the LP complex. The LP thus contains about 31% more neurons than the dLGN, and it occupies a volume 30% larger (0.35 ± 0.04 mm^3^ vs. 0.25 ± 0.02 mm^3^; as a result, neuron density is equivalent in both nuclei (**Figures 4B,C**).

Since cell density is roughly homogenous throughout the LP complex, given the difficulty of consistently delineating its LP nuclei borders on Nissl-stained sections, we attributed to each LP nucleus a number of neurons proportional to its measured volume. Accordingly, the LPMR was estimated to contain a mean of ∼15,400 total neurons, the LPL 12,200 total neurons and the LPMC ∼ 4,300 total neurons.

### Thalamic Interneuron Numbers

**FIGURE 5:**
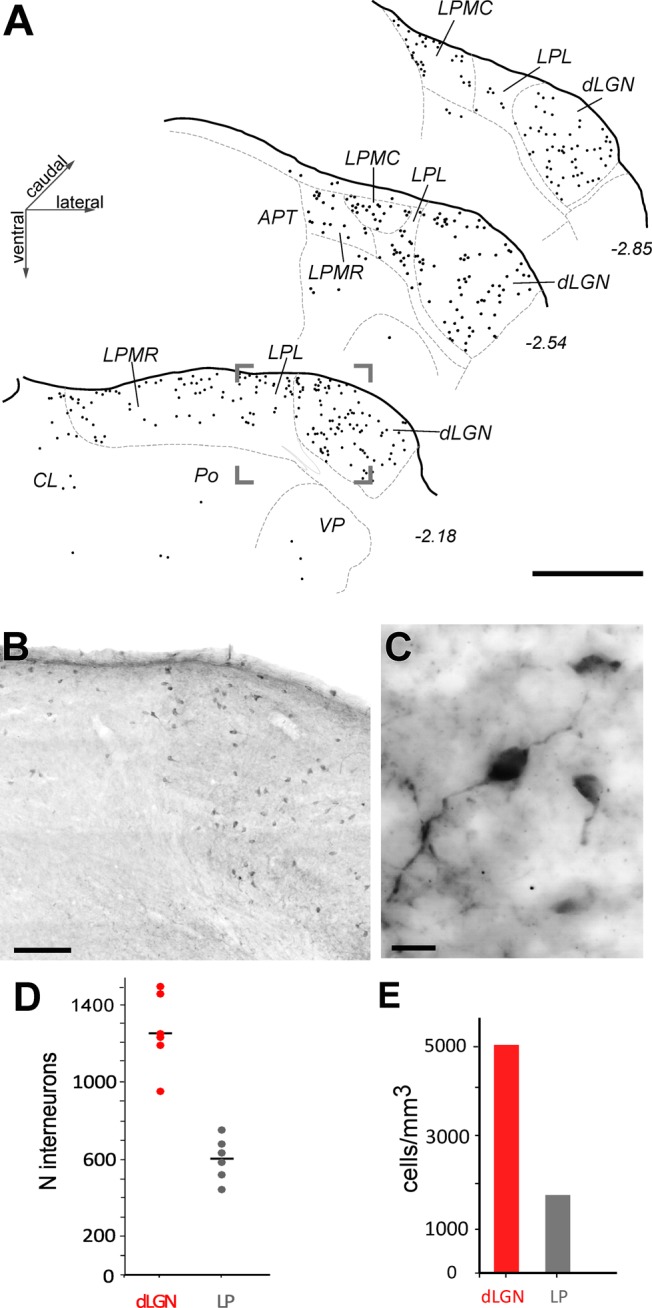
Distribution and number of GABAergic interneurons in the visual thalamic nuclei. **(A)** Camera-lucida drawings of three coronal sections of the visual thalamus. GABA-immunolabeled cells are indicated by black dots; each dot represents a cell. **(B)** Low-magnification image taken from the area indicated by a frame in **(A)**. **(C)** High magnification image showing three immunostained cells displaying typical interneuronal size and morphology. Scale bars: **(A)** = 500 μm; **(B)** = 50 μm; **(C)** = 10 μm. **(D,E)** GABAergic neuron number **(D)** and density **(E)** estimations.

To determine how many of the neurons in LP and dLGN are GABAergic interneurons, we immunolabeled for GABA and counted the labeled cells with the optical fractionator. A sizable population of GABA-positive cells was observed in dLGN and LP (**Figures 5A,B**). Occasional isolated GABA-immunoreactive cells with similar bipolar morphology were visible in most other dorsal thalamic nuclei. Some such cells in the posterior (Po) and ventroposterior nuclei (VP) are indicated in **Figure 5A**. The GABA-immunoreactive cells displayed typical thalamic interneuron morphologies (Jones, 2007): round, fusiform or triangular cell body (<10 μm transverse diameter) and few, and non-spinous proximal dendrites (**Figure 5C**).

The dLGN contained more GABA-immunoreactive cells (1,255 cells ± 194 SD) than the LP as a whole (606 cells ± 114 SD, **Figure 5D** and **Supplementary Table S3**). Interneuron density was 5,007 cells/mm^3^ in dLGN. Within LP, the GABA-immunoreactive interneurons were consistently concentrated in the more dorsal part of the complex, near the pial surface (**Figure 5B**).

Since GABA-ergic interneuron axons remain in the vicinity of the parent soma and thus presumably do not contribute to the thalamocortical projection (Jones, 2007), it can be estimated from the above data that the C57BL/6 adult male mouse dLGN contains ∼20,000 cortically projecting neurons. Likewise, the LP complex can be inferred to contain ∼30,400 thalamocortical projection neurons, of which ∼12,000 are in LPL, ∼15,200 in LPMR and ∼4,200 in LPMC.

## Discussion

By applying a combination of histological staining methods for consistent nuclear delineation and stereological methods for cell counting, we have determined the number of projection neurons and of GABA-expressing interneurons in the primary or “first order” (dLGN; Guillery and Sherman, 2002) and association or “higher-order” (LP complex) thalamic visual nuclei in the adult C57BL/6 male mouse. This dataset may be relevant both for constraining biologically accurate models of mouse thalamus and visual thalamocortical circuits (Hill and Tononi, 2004; Hawrylycz et al., 2017), as well as for quantitative comparisons with other mouse strains, or across species.

### Delimitation of LP Complex Divisions

Accurately counting neuron numbers on brain sections requires a consistent and precise delineation of the regions to be measured; this becomes most important when the regions are small in absolute terms, as the ones examined in the present study, because little delineation differences can then lead to substantially different results.

The mouse dLGN can be readily delineated on Nissl-stained sections. This is also the case with the LP complex as a whole. However, delineating the LP complex nuclei is more challenging. These nuclei have been primarily defined based on their afferent and efferent projection patterns, and their boundaries are not evident on Nissl-stained sections (Takahashi, 1985; Seabrook et al., 2017). Nevertheless, previous studies in the rat indicate that most such borders can be delineated from the comparison of the tissue staining patterns for several substances (Nakamura et al., 2015; Zhou et al., 2017). Here, we applied similar criteria to delineate the mouse nuclei.

We found the heavy VGluT2 immunostaining particularly informative for delineating LPMC. A lighter VGluT2 labeling is observed in LPL, while LPMR is essentially free of labeling (**Figures 1–3**). A heterogeneous distribution of the vGluT2-immunoreactive terminals in the three LP nuclei has been described in previous studies of visual thalamic nuclei of rats and non-rodent species (Chomsung et al., 2008; Nakamura et al., 2015; Zhou et al., 2017). Immunolabeling for VGluT2 selectively reveals the neurotransmitter vesicle pools inside glutamatergic terminals of spinal cord, brainstem, retina and diencephalon origin. In contrast, VGluT2 is absent from glutamatergic axons of cortical origin, such as those of corticothalamic axon terminals (Fujiyama et al., 2001; Groh et al., 2014; Bopp et al., 2017). The size and appearance of the dLGN VGluT2+ terminals are congruent with that of the axon terminals from retinal ganglion cells (Dursun et al., 2013; Hammer et al., 2014; Hong et al., 2014). The small boutons in LP might correspond to retinal ganglion cell axons, particularly those in LPMR or the anterior part of LPL, where thinner and sparser retinal axon arborizations have been labeled by choleratoxin B subunit eye injections (Morin and Studholme, 2014). On the other hand, the large VGluT2+ boutons present in the LPMC and LPL probably correspond to the terminals from axons originated in the superficial layers of the superior colliculus (Masterson et al., 2009; Bickford et al., 2015; Baldwin et al., 2017).

In consonance with observations in the rat by Résibois and Rogers (1992), Winsky et al. (1992), Arai et al. (1994), and Zhou et al. (2017), we also observed differences regarding the CR labeling in the three LP nuclei (**Figures 1, 2**). This pattern is quite helpful for separating LPMR and LPMC from LPL.

### Total Neuron Numbers in Mouse Visual Thalamic Nuclei

We estimated the total neuronal number in dLGN and the various LP nuclei using an unbiased stereological design. Our numbers are consistent with that of Seecharan et al. (2003) for C57BL/6 mice dLGN (∼21,200 vs. 19,900; a mere 6% mean value difference), despite the fact that: (a) we examined, for maximal consistency, only young adult (60- to 80-day-old) males instead of a mixture of males and females of a much wider age spectrum (35–694 days) as Seecharan et al. (2003) reportedly did; and (b) both studies were conducted by independent researchers delineating the nuclei on Nissl-stained sections and applying different stereological setups and algorithms. It is to be noted that the Seecharan et al. (2003) study revealed a surprising range (up to 30%) of variation in dLGN neuron numbers between inbred mice strains, and showed C57BL/6 to be one of the strains with more numerous dLGN neurons. Such differences may have contributed to lower neuron estimates in previous, classical non-stereological studies that were conducted on other mouse strains (Cullen and Kaiserman-Abramof, 1976; Heumann and Rabinowicz, 1980).

This is, to our knowledge, the first published estimate of total neuron number of LP in a rodent. Our data show that the mouse LP complex as a whole contains ∼31,000 neurons, about one third more than dLGN. While the dLGN neurons innervate almost exclusively area V1, the LP cells innervate both V1 and its surrounding secondary visual areas (Sanderson et al., 1991; Tohmi et al., 2014). Cells in the medial parts of LP innervate also multimodal temporal and medial limbic cortical areas, and even the striatum. Many of these widely spread LP connections may be established through collateral branches of the same cell axon (Nakamura et al., 2015).

Based on its afferent and efferent connections, the LP is regarded as the rodent equivalent of the primate pulvinar complex (Baldwin et al., 2017; Zhou et al., 2017). Thus, the present data may be a relevant not only for modeling and inter-species comparisons of first-order visual thalamocortical circuits, but also of “higher-order” visual thalamocortical pathways (Sherman and Guillery, 2002).

### GABA-ergic Interneuron Numbers in Mouse Visual Thalamic Nuclei

In addition to thalamocortical projection neurons, the mouse visual thalamus was known to contain a sizable population of GABAergic local-axon neurons (Arcelli et al., 1997; Jones, 2007; Jager et al., 2016; Kerschensteiner and Guido, 2017). However, the number of these cells and their prevalence had not previously been examined with unbiased quantitative methods.

Our data show that GABA-immunopositive interneurons are just 5.8% of the total dLGN neurons. Published estimates of the prevalence of interneurons within dLGN are higher: 8% (based on Golgi staining and two-dimensional counting, Werner et al., 1984) or 15–20% (based on GABA immunostaining + thionin two-dimensional counting, Arcelli et al., 1997). We are confident that the difference with our results reflects the accuracy of the stereological counting procedure, not a less-sensitive immunolabeling, for several reasons. First, we applied antigen retrieval, up to 20-fold higher primary antibody concentrations than those used by Arcelli et al. (1997), and long (48 h) antibody incubations. Second, cell labeling in our material was sharp and complete throughout the whole thickness of the sections (**Figure 5** and **Supplementary Figure S2**). Third, the observed distribution and abundance of neurons matches that illustrated in previous GABA immunolabeling studies in mice and other rodents (Ohara et al., 1983; Ottersen and Storm-Mathisen, 1984; Spreafico et al., 1994; Arcelli et al., 1997; Jager et al., 2016), as well as the glutamate decarboxylase 1 (GAD1) mRNA expression pattern (Lein et al., 2007; Allen Brain ISH bank, experiment #479); and, finally, our numbers are virtually identical (1.255 cells vs. 1.300 cells) to those reported by Golding et al. (2014) who directly counted every cell, one by one, within the dLGN of P21 GAD67-GFP transgenic mice.

No previous estimates of GABAergic interneuron prevalence in rodent LP are available. Our data show these cells are quite scarce in the LP complex overall (1.9%). The labeling pattern again matches the GAD1 *in situ* hybridization data of Lein et al. (2007). Neurons tend to be more abundant in the dorsal part of the nuclei, near the pia, a pattern that might result from the subpial dispersal of neurons migrating into these nuclei from adjacent prosomeres during development (Golding et al., 2014; Jager et al., 2016). Early circuit activity of the retinal afferent terminals is believed to influence this migration (Li et al., 2003; López-Bendito and Molnár, 2003; Golding et al., 2014).

In any case, our data demonstrate that GABA interneurons are scarcer in the mouse visual thalamus than previously assumed. This is in striking contrast with visual thalamic nuclei of carnivores and primates, where GABA-ergic interneurons reportedly represent 20–25% of all neurons (Hunt et al., 1991; Arcelli et al., 1997; Jones, 2007).

### Volume of dLGN and LP Complex

We measured the absolute volume of the various visual thalamus nuclei. We then compared these measurements with the numbers of neurons in each, to calculate neuronal density. We found that cell density in dLGN is similar to the neuron density in LP. We observed no differences in absolute neuron numbers between the same visual nuclei of the left and right thalami. Likewise, we did not find differences in volume between left and right LP; however, we found that left dLGN volume was ∼7% larger in all our animals, a statistically significant difference (*p* = 0.039). Interhemispheric differences in cell number and tissue volume have been previously observed in the rodent somatosensory cortex, and related to a lateralized use of sensory systems (Tobet et al., 1993; Machín et al., 2004). It is conceivable that a larger LGN volume might similarly reflect neuropil changes associated with some degree of lateralized use or dominance of the retinogeniculate pathways (Treviño, 2014).

## Author Contributions

ME prepared the histological material, carried out the stereological analysis, made figures, and co-wrote the paper. MG-A designed and carried out the stereological analysis and co-wrote the paper. FC designed the study and co-wrote the paper.

## Conflict of Interest Statement

The authors declare that the research was conducted in the absence of any commercial or financial relationships that could be construed as a potential conflict of interest.

## Supplementary Material

FIGURE S1 Stereological methods used for estimating volume **(A,B)** and neuron number **(C,D)** in dLGN and LP complex on Nissl-stained coronal sections. **(A,B)** Delimitation of dLGN (red) and LP complex (blue). An ideal counting frame is shown in each section where it is identified the exclusion (red) and inclusion (green) borders. In **(A)** is also shown the point grid (green) used to estimate the nuclear volume with the Cavalieri method. **(C,D)** Representative microphotographs of coronal section showing a representative unbiased counting frame. Neurons (red arrows) are identified by their darkly stained nucleolus in a lighter stained nucleus and their large size; glial cells (green arrows) are much smaller, have multiple nucleoli and no visible cytoplasm; endothelial cells (blue arrows) have a curved elongated shape and usually no visible nucleolus. Scale bars **A, B** = 100 μm; **C, D** = 10 μm.

FIGURE S2 Complete penetration of the GABA antibody in the tissue sections. **(A–C)** Examples of neurons immunostained for GABA. The soma of these neurons was located in the center of the sections (*z* = 6–8 μm on the dried, mounted sections).

TABLE S1 Total neuron number estimations in visual thalamic nuclei.

TABLE S2 Volume estimations of the visual thalamic nuclei (in mm^3^) and interhemispheric differences, (in mm^3^).

TABLE S3 Number of GABAergic interneurons in visual thalamic nuclei.

## Abbreviations

AChE: acetylcholinesterase
APT: anterior pretectal- nucleus
bsc: brachium of the superior colliculus
CB: calbindin
CE: error coefficient
CL: central lateral thalamic nucleus
CR: calretinin
ctt: corticotectal fiber bundles
CyO: cytochrome oxidase
dLGN: dorsal lateral geniculate nucleus
eml: external medullary lamina
IGL: intergeniculate leaflet
ima: intrathalamic medullary area
LD: laterodorsal thalamic nucleus
LP: lateroposterior complex of the thalamus
LPL: lateral part of the lateroposterior thalamic complex
LPMC: mediocaudal lateroposterior thalamic nucleus
LPMR: mediorrostral lateroposterior thalamic nucleus
MGN: medial geniculate nucleus
N: total neuron number
ot: optic tract fibers
Po: posterior thalamic nucleus
V: nuclear volume
VGluT2: vesicular glutamate transporter type 2
VP: ventral posterior thalamic nucleus.
