# Supplementary figures and images for: Thalamocortical Projection Neuron and Interneuron Numbers in the Visual Thalamic Nuclei of the Adult C57BL/6 Mouse
Source: Front Neuroanat. 2018 Apr 12;12:27. doi: 10.3389/fnana.2018.00027 (PMC5906714; doi:10.3389/fnana.2018.00027)

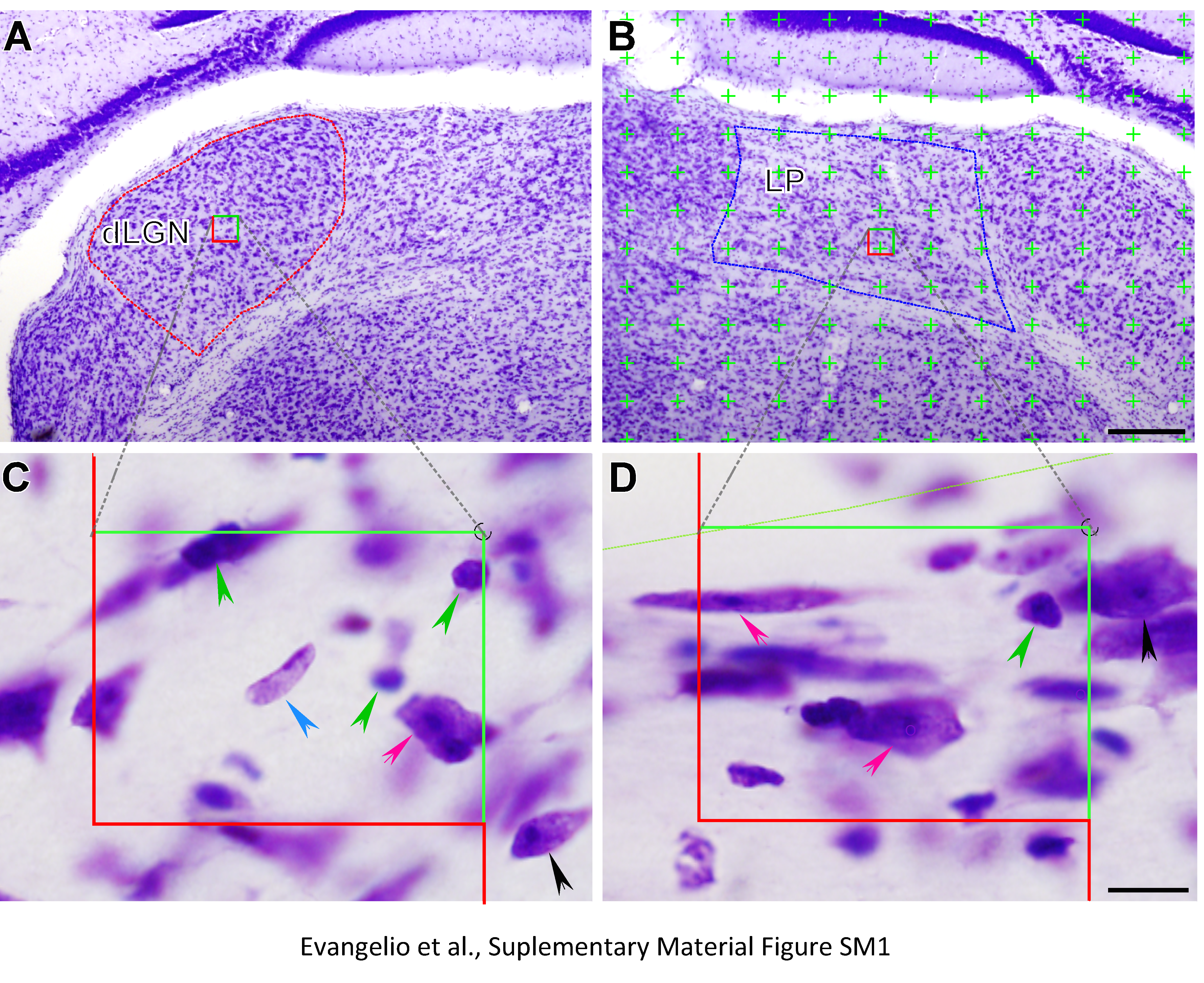

Supplement: FIGURE S1 — Stereological methods used for estimating volume (A,B) and neuron number (C,D) in dLGN and LP complex on Nissl-stained coronal sections. (A,B) Delimitation of dLGN (red) and LP complex (blue). An ideal counting frame is shown in each section where it is identified the exclusion (red) and inclusion (green) borders. In (A) is also shown the point grid (green) used to estimate the nuclear volume with the Cavalieri method. (C,D) Representative microphotographs of coronal section showing a representative unbiased counting frame. Neurons (red arrows) are identified by their darkly stained nucleolus in a lighter stained nucleus and their large size; glial cells (green arrows) are much smaller, have multiple nucleoli and no visible cytoplasm; endothelial cells (blue arrows) have a curved elongated shape and usually no visible nucleolus. Scale bars A, B = 100 μm; C, D = 10 μm. [file Image_1.TIF]

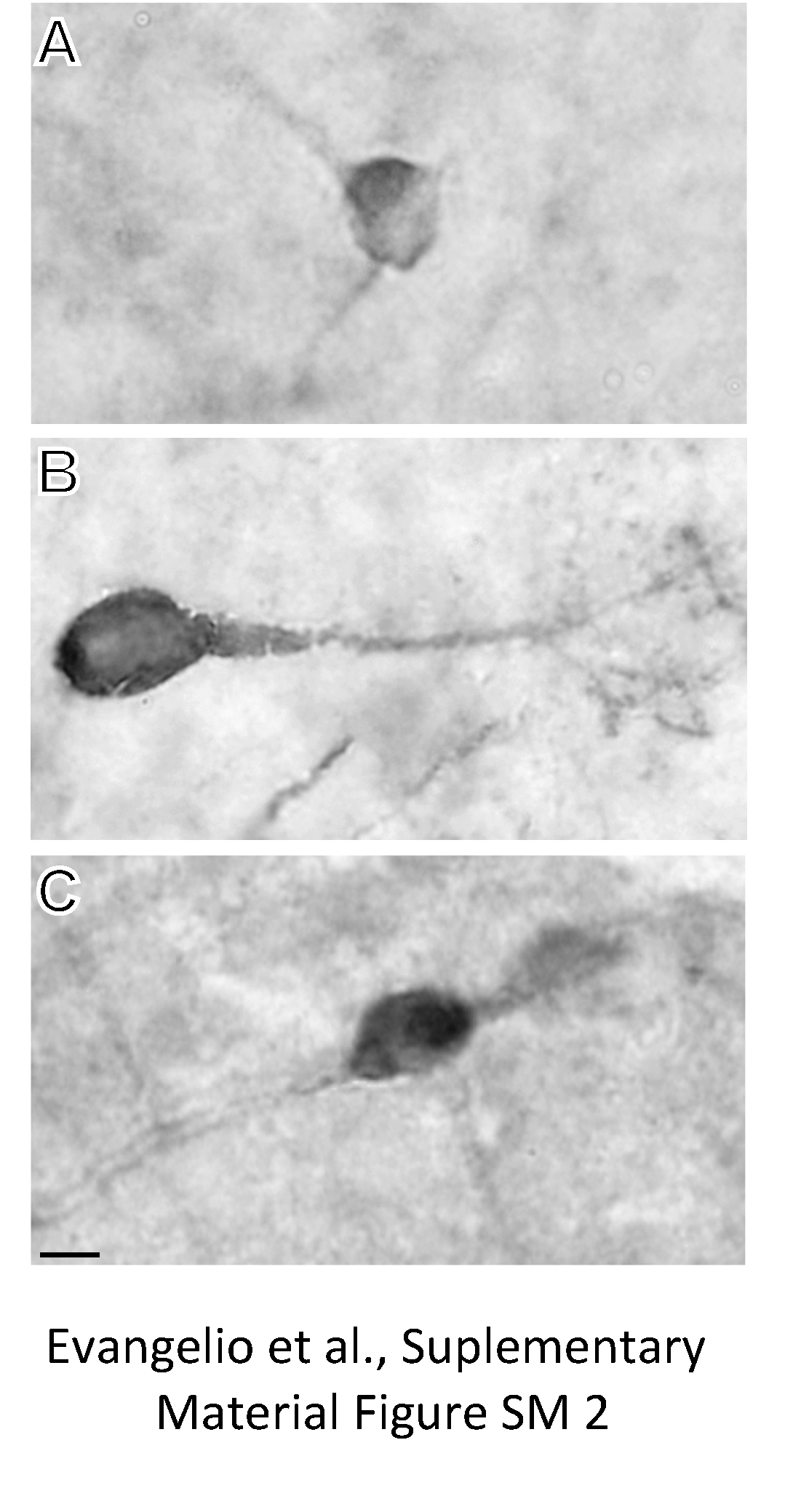

Supplement: FIGURE S2 — Complete penetration of the GABA antibody in the tissue sections. (A–C) Examples of neurons immunostained for GABA. The soma of these neurons was located in the center of the sections (z = 6–8 μm on the dried, mounted sections). [file Image_2.TIF]
