# Supplementary material for: Thalamocortical Projection Neuron and Interneuron Numbers in the Visual Thalamic Nuclei of the Adult C57BL/6 Mouse
Source: Front Neuroanat. 2018 Apr 12;12:27. doi: 10.3389/fnana.2018.00027 (PMC5906714; doi:10.3389/fnana.2018.00027)
Supplement: TABLE S1 — Total neuron number estimations in visual thalamic nuclei. [file Table_1.docx]

**TABLE SM1. Total neuron number estimations in visual thalamic nuclei**

| *Case* | *Hemisph* | *dLGN* | *LP (total)* |
| --- | --- | --- | --- |
| R1 | R | 18,678 | 30,536 |
| R1 | L | 19,912 | 30,536 |
| R2 | R | 18,630 | 35,107 |
| R2 | L | 18,954 | 31,630 |
| R3 | R | 23,980 | 32,606 |
| R3 | L | 23,462 | 37,757 |
| R4 | R | 20,920 | 26,150 |
| R4 | L | 20,049 | 26,586 |
| R5 | R | 23,571 | 33,460 |
| R5 | L | 23,771 | 26,168 |
| *Mean N* |  | **21,193** | **31,054** |
| *SD* |  | 2,264 | 3,920 |
| *Mean CE* |  | 0.091 | 0.070 |

*SD, standard deviation; CE, coefficient of error; R, right; L, left.
