# Supplementary material for: Thalamocortical Projection Neuron and Interneuron Numbers in the Visual Thalamic Nuclei of the Adult C57BL/6 Mouse
Source: Front Neuroanat. 2018 Apr 12;12:27. doi: 10.3389/fnana.2018.00027 (PMC5906714; doi:10.3389/fnana.2018.00027)
Supplement: TABLE S2 — Volume estimations of the visual thalamic nuclei (in mm3) and interhemispheric differences, (in mm3). [file Table_2.docx]

**TABLE SM2.** V**olume estimations of the visual thalamic nuclei (mm^3^)**

| Case | Hemisph | dLGN | LP (total) |
| --- | --- | --- | --- |
| R1 | R | 0.231 | 0.414 |
| R1 | L | 0.242 | 0.388 |
| R2 | R | 0.248 | 0.389 |
| R2 | L | 0.279 | 0.399 |
| R3 | R | 0.270 | 0.343 |
| R3 | L | 0.288 | 0.361 |
| R4 | R | 0.225 | 0.308 |
| R4 | L | 0.246 | 0.315 |
| R5 | R | 0.232 | 0.312 |
| R5 | L | 0.245 | 0.321 |
| *Mean V* |  | **0.251** | **0.355** |
| *SD* |  | 0.021 | 0.04 |
| *Mean CE* |  | 0.079 | 0.063 |
|  |  |  |  |

**Interhemispheric differences (in mm^3^)**

|  | dLGN | LP (total) |
| --- | --- | --- |
| *Mean V R* | 0.241 | 0.353 |
| *Mean V L* | 0.260 | 0.357 |
| Diff | 0.019* | 0.004 |

*SD, standard deviation, CE, coefficient of error; Diff, difference; R, right; L, left.
