# Supplementary material for: Thalamocortical Projection Neuron and Interneuron Numbers in the Visual Thalamic Nuclei of the Adult C57BL/6 Mouse
Source: Front Neuroanat. 2018 Apr 12;12:27. doi: 10.3389/fnana.2018.00027 (PMC5906714; doi:10.3389/fnana.2018.00027)
Supplement: TABLE S3 — Number of GABAergic interneurons in visual thalamic nuclei. [file Table_3.docx]

**TABLE SM3. Number of GABAergic interneurons in visual thalamic nuclei**

| *Case* | *Hemisph* | *dLGN* | *LP* |
| --- | --- | --- | --- |
| R1 | R | 1,228 | 443 |
| R1 | L | 1,431 | 526 |
| R2 | R | 1,495 | 634 |
| R2 | L | 1,238 | 761 |
| R3 | R | 1,190 | 685 |
| R3 | L | 947 | 585 |
| *Mean N* |  | **1255** | **606** |
| *SD* |  | 194 | 114 |
| *Mean CE* |  | 0.069 | 0.190 |
| Total neurons |  | 21,193 | 31,054 |
| Projection neurons |  | 19,938 | 30,448 |

*SD, standard deviation; CE, coefficient of error; R, right; L, left.
